# The Role of Vitamin E Isoforms and Metabolites in Cancer Prevention: Mechanistic Insights into Sphingolipid Metabolism Modulation

**DOI:** 10.3390/nu16234115

**Published:** 2024-11-28

**Authors:** Yumi Jang, Choon Young Kim

**Affiliations:** 1 Department of Food Science and Nutrition, University of Ulsan, Ulsan 44610, Republic of Korea; yjang@ulsan.ac.kr; 2 Basic-Clinical Convergence Research Institute, University of Ulsan, Ulsan 44610, Republic of Korea; 3 Department of Food and Nutrition, Yeungnam University, Gyeongsan 38541, Republic of Korea; 4 Research Institute of Human Ecology, Yeungnam University, Gyeongsan 38541, Republic of Korea

**Keywords:** cancer, tocopherols, tocotrienols, metabolite, 13′-carboxychromanol, sphingolipids

## Abstract

Natural forms of vitamin E include four tocopherols and four tocotrienols (α, β, γ, and δ), which are essential as lipophilic antioxidants. Among these eight isoforms, α-tocopherol (αT), the predominant form of vitamin E found in tissues, has traditionally received the most attention in disease prevention research due to its robust antioxidant activity. However, recent studies suggest that other forms of vitamin E exhibit distinct and potentially more potent beneficial activities in disease prevention and treatment. These non-αT forms of vitamin E are metabolized in vivo, producing various metabolites, including 13′-carboxychromanol, though their biological roles remain largely unknown. Notably, sphingolipids, known for their significant roles in cancer biology, may be involved in the anticancer effects of vitamin E through the modulation of sphingolipid metabolism. This review focuses on the diverse biological activities of different vitamin E forms and their metabolites, particularly their anticancer effects, while highlighting the underlying mechanisms, including their novel impact on regulating sphingolipid pathways. By elucidating these interactions, we aim to provide a deeper understanding on the multifaceted roles of vitamin E in cancer prevention and therapy.

## 1. Introduction

The research on vitamin E, including α-, β-, γ-, δ-tocopherols and α-, β-, γ-, δ-tocotrienols for disease prevention and therapy has primarily concentrated on α-tocopherol (αT), recognized as the most potent antioxidant among vitamin E forms. However, despite being the predominant form of vitamin E in tissues, αT has demonstrated inconsistent and sometimes disappointing results in various epidemiological studies, particularly those related to cancer prevention. In contrast, other vitamin E forms, collectively termed non-αT forms, exhibit low concentrations in tissues due to extensive in vivo metabolism. Nevertheless, these non-αT forms have demonstrated robust anticancer activities in both in vivo and in vitro studies. The metabolism of vitamin E forms results in the production of long- and short-chain hydroxychromanols and carboxychromanols. Among these metabolites, 13′-carboxychrmanol (13′-COOH) has recently emerged as the most abundant metabolite in feces, displaying novel and potent disease-preventing effects. However, there is a lack of well-established knowledge regarding the biological activities and functions of these long-chain metabolites. This review aims to consolidate the current understanding of natural vitamin E forms and their metabolites, exploring their underlying mechanisms, particularly their anticancer effects. Emphasis is placed on their modulation of sphingolipid metabolism, representing a novel anticancer mechanism.

## 2. Vitamin E

### 2.1. Vitamin E Forms and Food Sources

**Figure 1:**
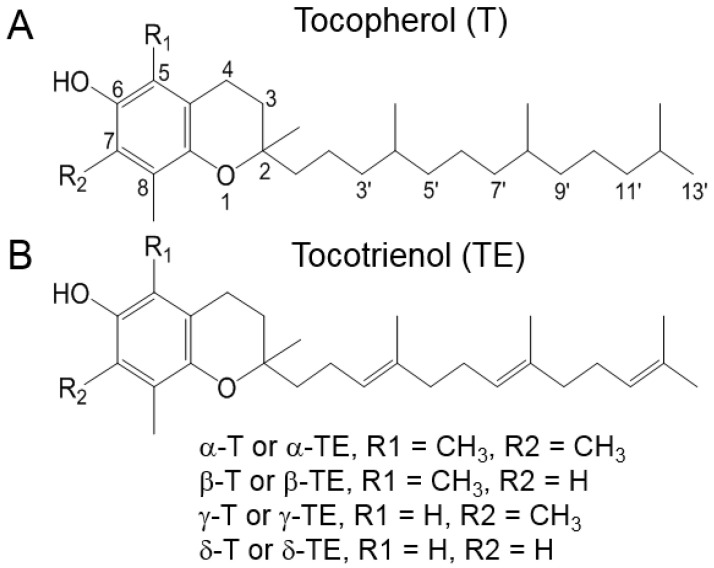
Structures of natural forms of vitamin E. (**A**) Tocopherols and (**B**) tocotrienols. All vitamin E forms contain a 16-carbon phytyl chain attached to the 2-position of a chromane ring. The difference between tocopherols and tocotrienols is that tocotrienols have an unsaturated phytyl chain with three double bonds.

Vitamin E is a crucial nutrient and a well-known lipophilic chain-breaking antioxidant. In 1922, Evans and Bishop first identified vitamin E as an essential dietary factor for reproduction in rats [1]. Since this discovery, numerous studies have focused on the potential health benefits of vitamin E for various diseases. Natural forms of vitamin E consist of eight structurally related compounds, which can be further categorized into two main groups: tocopherols and tocotrienols. Both tocopherols and tocotrienols share the same basic chemical structure, characterized by a 16-carbon phytyl chain attached to the 2-position of a chromane ring. The primary difference between the two is that tocopherols have a saturated phytyl chain, while tocotrienols feature an unsaturated phytyl chain with three double bonds. The individual tocopherols and tocotrienols—namely α-, β-, γ-, and δ-tocopherol (αT, βT, γT, and δT) and α-, β-, γ-, and δ-tocotrienol (αTE, βTE, γTE, and δTE)—differ from each other based on the number and position of methyl groups on the chromane ring. The structures of tocopherols and tocotrienols are shown in Figure 1.

Furthermore, vitamin E is found in both natural and synthetic forms. Natural forms of tocopherol have the RRR configuration at the 2, 4′, and 8′-positions of the phytyl tail, while natural forms of tocotrienol have the R configuration at the 2-position of the phytyl tail. Both the natural single stereoisomeric form of α-tocopherol (RRR, formerly *d*-α-tocopherol) and synthetic form of α-tocopherol are available on the market as dietary supplements. When α-tocopherol is synthesized chemically, this synthetic form consists of an approximately equimolar mixture of all eight possible stereoisomers (all-racemic α-tocopherol, formerly *dl*-α-tocopherol): RRR, RRS, RSS, RSR, SRR, SSR, SRS, and SSS [2,3]. Although they have similar structures, the different stereoisomers of vitamin E have different bioavailability and bioactivity, which will be discussed in the next section. In addition, because the free forms of vitamin E are easily oxidized, more stable forms of vitamin E have been produced for the use as dietary supplements. Since ester forms are very stable, α-tocopherol is usually sold either as acetate esters (RRR α-tocopheryl acetate or all-rac α-tocopheryl acetate) or as succinate esters (RRR α-tocopheryl succinate or all-rac α-tocopheryl succinate). Among these, the acetate ester of all-rac α-tocopherol is the most common form of vitamin E supplementation due to its cost-effectiveness and the stability [4].

Since humans and animals do not produce vitamin E, they acquire natural forms of vitamin E from plants. Vitamin E is found in various foods, such as some fruits, vegetable oils, green leafy vegetables, plant seeds, corn, barley, oats, and wheat germ. Natural sources of tocopherols include vegetable oils, plant seeds, and nuts. For instance, peanuts, almonds, and sunflower seeds are major sources of αT, while γT is found in walnuts, pecans, corn oil, soybean oil, and sesame oil. δT is found in soybean oil and rice germ. Since corn and soybean oils are widely used, γT accounts for about 70% of the vitamin E in the US diet. On the other hand, tocotrienols are mainly found in palm oil, barley, oats, and some cereal grains, but in much smaller amounts than tocopherols [5,6,7].

### 2.2. Bioavailability and Metabolites

Vitamin E is a lipophilic (fat-soluble) vitamin, and similar to other lipids, all forms of vitamin E are absorbed equally in the intestine along with dietary fat and incorporated into chylomicrons. The chylomicron-bound vitamin E is transported through the lymphatic system to peripheral tissues such as the skin, adipose tissue, muscle, or brain with the help of lipoprotein lipase. The chylomicron remnants are then taken up by the liver for metabolism or further distribution. In the liver, αT is preferentially incorporated into very-low-density lipoproteins due to the high affinity of α-tocopherol transfer protein (α-TTP) and the ATP-binding cassette transporter A1 (ABCA1), and is redistributed throughout the body. Therefore, αT is the predominant form in most human and animal tissues and plasma, as it is protected by α-TTP from being metabolized [3,7,8,9,10].

**Figure 2:**
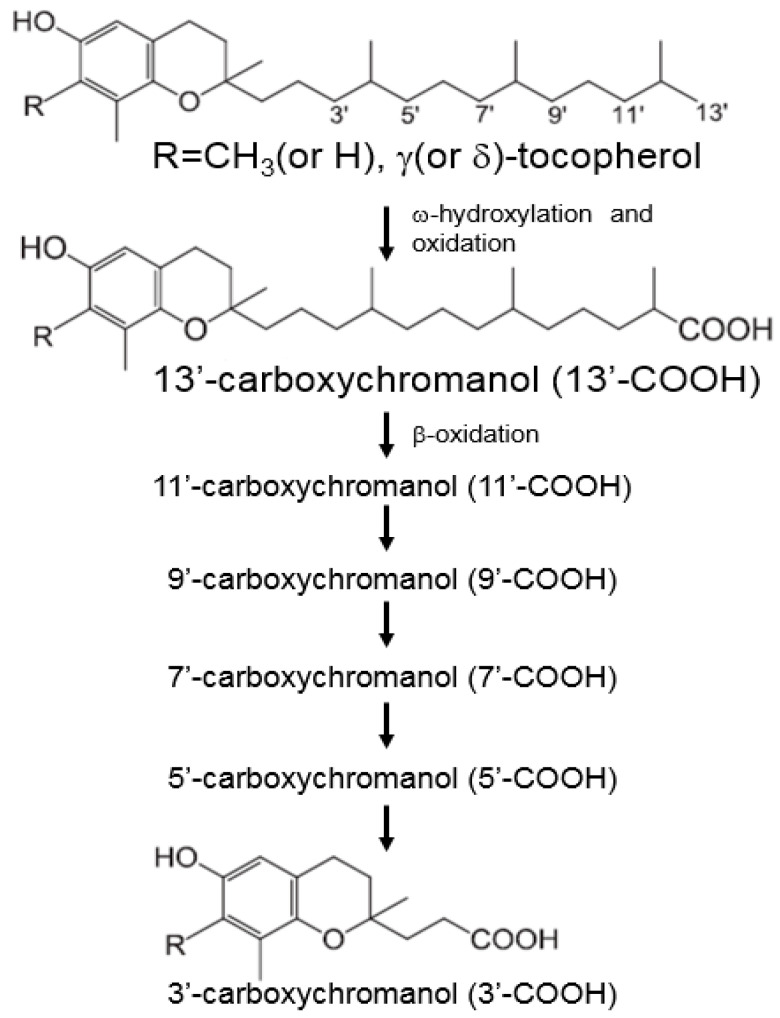
Metabolism of vitamin E forms and their metabolites. Vitamin E forms are metabolized via ω-hydroxylation and β-oxidation.

In contrast, α-TTP has much lower affinity for other forms of vitamin E. As a result, non-αT forms of vitamin E such as β-, γ-, δ-T and α-, β-, γ-, δ-TE are relatively low in tissues, as they are preferentially catabolized in the liver via cytochrome P450 (CYP4F2)-mediated ω-hydroxylation to 13′-hydroxychromanol (13′-OH). 13′-OH is then further oxidized to 13′-carboxychromanol (13′-COOH), followed by β-oxidation of the phytyl chain to generate various shorter chain-length carboxychromanols, including 11′- and 9′-COOHs, and the terminal product 3′-COOH or (2′-carboxyethyl)-6-hydroxychromans (CEHCs). CEHCs are the water-soluble metabolites of vitamin E and are primarily excreted out of the body via the urine (Figure 2) [8,11,12]. In parallel with β-oxidation, conjugation reactions such as sulfation and glucuronidation of the phenolic group on the chromane ring occur in the intermediate metabolites when the intake of vitamin E forms is high [13]. Therefore, despite γT being the major form of vitamin E in the US diet, αT is the predominant form of vitamin E in the body. The plasma concentrations of αT are about 20~30 μM, but γT is 5–10 times lower than αT in the plasma [7,14]. Additionally, the bioavailability of γT is suppressed by increased intake of αT [15], but γT supplementation has been shown to increase the levels of both tocopherols [16].

In addition to the different bioavailability of each natural vitamin E form, the different stereoisomers of vitamin E also have varying bioavailability and biopotency. Both natural and synthetic vitamin E are absorbed in the body. However, after absorption, RRR-αT, a natural form of vitamin E, has a greater bioavailability and activity than the all-rac forms due to the higher affinity of RRR-αT toward the liver protein α-TTP, compared to the synthetic forms. Thus, synthetic forms of vitamin E are preferentially excreted. The bioavailability of natural vitamin E is about twice as high as that of synthetic forms, meaning that twice the amount of synthetic forms of vitamin E is required to match the bioavailability of the natural forms [2,3,4].

### 2.3. Bioactivities of Vitamin E

The biological activity of vitamin E compounds has historically been assessed using the rat fetal resorption assay. In this assay, the biological activity of vitamin E is measured based on its ability to prevent or reverse the symptoms of vitamin E deficiency, such as fetal resorption, encephalomalacia, muscular dystrophy, and subsequent embryo death. Among the natural forms of vitamin E, αT has been shown to have the highest biological activity as measured by this assay. Studies have also demonstrated that α-TTP is present in the pregnant rodent uterus and that αT is important during pregnancy [17]. However, the difference in activity between vitamin E forms appears to be caused by the lower concentrations of non-αT forms of vitamin E in tissues and plasma, due to their shorter retention time, rather than differences in their actual biological activity.

#### 2.3.1. Antioxidant Function

Free radicals generated by oxidative stress play an important role in the development of numerous diseases, including cancer, atherosclerosis, and neurodegenerative diseases. All vitamin E forms have been well recognized as one of the most important antioxidants. It is known to be a potent, lipophilic peroxyl radical scavenger that protects biological molecules from oxygen toxicity. In vitamin E studies, most research has primarily focused on αT until recently, because αT is the predominant form of vitamin E in tissues and plasma, and due to its highest biological activities as well as the relationship between its low intake and the higher incidence of vitamin E deficiency-associated ataxia. Moreover, among tocopherols, αT has been shown to have the highest antioxidant activities against peroxyl radical (LOO·) and delta-tocopherol is the least active (alpha > beta = gamma > delta). αT terminates the chain reaction of free radical mediated lipid peroxidation by donating hydrogens from the phenolic group on the chromane ring to lipid radicals. Therefore, αT protects lipid membrane from lipid radical damage [18]. After αT becomes a free radical itself, it can be recycled back to antioxidant form by vitamin C (ascorbic acid) [19]. In contrast, γT has its unique ability to change highly reactive nitrogen back into safe NO, thereby protecting against oxidation damage by reactive nitrogen species. αT lacks this ability [7,20]. On the other hand, tocotrienols also show antioxidant effects by scavenging the chain-propagating peroxyl radical. It has been demonstrated that αTE has a more potent antioxidant activity than αT for the scavenging peroxyl radicals in liposomes as it is distributed more evenly in the phospholipid bilayer of the plasma membrane, and it shows more efficient collision with radicals [21,22]. In addition to tocopherols and tocotrienols, 13′-carboxychromanols from δT and δTE, which are long-chain metabolites of vitamin E, have been shown to exert stronger antioxidative activities compared with *dl*-αT [8,23].

#### 2.3.2. Non-Antioxidant Functions

**Figure 3:**
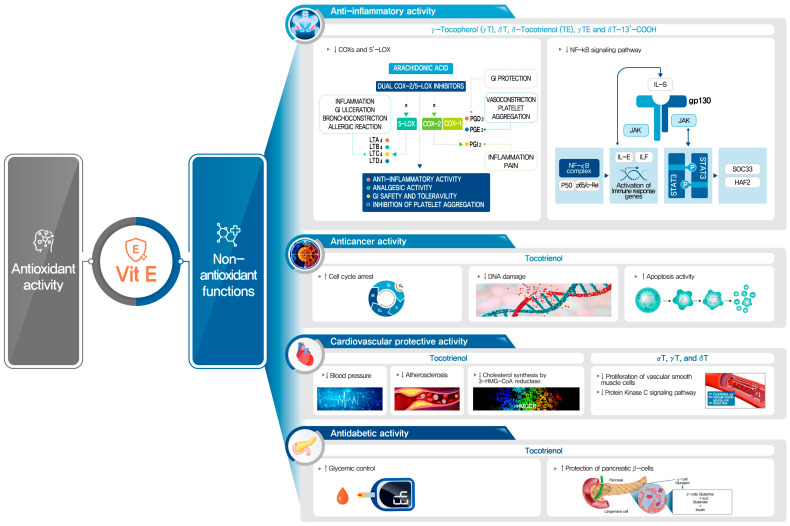
Non-antioxidant activities of vitamin E forms and their metabolites.

Besides antioxidant activities of vitamin E, vitamin E isoforms have other biological functions independent of their antioxidant properties such as anti-inflammatory, anticancer, cardioprotective, and antidiabetic effects (Figure 3). Chronic inflammation has been recognized as contributing to the development of other chronic diseases including cardiovascular diseases and cancer [24,25]. Eicosanoids derived from arachidonic acid via cyclooxygenases (COX) and 5-lipoxygenase (LOX)-catalyzed reactions play important roles in the regulation of inflammation and cancer [26,27]. Mechanistic studies have shown that vitamin E forms such as γT, δT, and γTE exert anti-inflammatory activities by inhibiting COXs and 5-LOX activities and suppressing nuclear factor κB (NF-κB) and Janus kinase (JAK)-signal transducer and activator of transcription (STAT) 6 or JAK-STAT3 signaling pathways in various types of cells [8,22,28,29]. In particular, novel mechanisms of the anti-inflammatory effects of γTE have been discovered, in which γTE inhibits TNFα-stimulated NF-κB, TAK1 and JNK activation by modulating sphingolipids and inducing ER stress, followed by the up-regulation of A20 [30]. In addition, δT-13′-COOH, a long-chain carboxychromanol from δT, has been demonstrated to competitively inhibit COX-1 and -2 activities, exhibiting much stronger effects than short-chain carboxychromanols and unmetabolized vitamin E forms [31]. δT-13′-COOH also inhibited 5-LOX activity [32], indicating that δT-13′-COOH is a unique dual inhibitor of COX-1/COX-2 and 5-LOX. In addition to the anti-inflammatory activities of vitamin E forms, αT, γT, and δT have been demonstrated to prevent the development of atherosclerosis by inhibiting protein kinase C activity and the proliferation of smooth muscle cell [33,34]. Several studies have shown that the plasma concentrations and the intake of γT are inversely associated with increased risk of cardiovascular disease (CVD), suggesting that γT is important in the defense against CVD [35]. Moreover, tocotrienols have been shown to have cardioprotective effects, which may stem from their blood-pressure-lowering, cholesterol-lowering, and antiatherogenic effects [22]. In the cholesterol-lowering activities of tocotrienols, tocotrienols affect the mevalonate pathway by post-transcriptionally suppressing the 3-hydroxyl-3-methyl-glutaryl CoA reductase [36].

Vitamin E forms also showed antidiabetic effects. For example, γT, but not αT, partially protected insulin-secreting cells from nitric oxide-induced functional inhibition [37] and palm vitamin E (tocotrienol-rich diet; TRF) decreased advanced glycosylation end points in non-diabetic rats and improved glycemic control in streptozotocin-induced diabetic rats [38].

Independent of its antioxidant activity, vitamin E forms also have shown to prevent cancer by inhibition of the cell-cycle, suppression of DNA synthesis and by inducing apoptosis [22]. The anticancer activities of each vitamin E form will be further discussed in the following section.

## 3. Different Forms of Vitamin E for Cancer Prevention

### 3.1. Mechanisms of Cancer Prevention

Vitamin E, which includes various isoforms such as tocopherols and tocotrienols, exhibits significant anticancer properties through multiple mechanisms. Since different forms of vitamin E and their metabolites show distinct anticancer activities, we will discuss the various actions underlying their anticancer effects in this following section. The anticancer mechanisms of vitamin E primarily involve anti-inflammatory effects [8,22,28,29,30,31,39], antioxidant activities [7,8,20,21,22,23], induction of apoptosis [40,41,42,43,44,45,46,47,48,49], cell cycle arrest [50,51], and modulation of cell signaling pathways [41,52,53,54].

### 3.2. Anticancer Effects and Mechanisms of Various Forms of Vitamin E

#### 3.2.1. α-Tocopherol in Cancer Prevention

Despite eight different forms of vitamin E, most studies have focused on αT for the last few decades due to its abundance in the body and robust antioxidant properties. In particular, all human intervention studies for vitamin E in cancer prevention have exclusively focused on αT. Since 1993, eight large randomized clinical trials (RCTs) have been conducted to examine the effect of αT or combinations of αT with other nutrients on cancer incidence and cancer mortality. The Linxian study [55] in China, which targeted a population with moderate micronutrient deficiencies, found that a combination of αT, β-carotene, and selenium reduced total cancer incidence and mortality, particularly in participants with low baseline nutrient intake. Similarly, the αT, β-Carotene Cancer Prevention Study (ATBC) [56] conducted in male smokers showed no effect on lung cancer, but suggested that αT could reduce the risk of prostate cancer, indicating that αT may be beneficial in high-risk individuals. The Heart Protection Study (HPS) [57], although focused on cardiovascular disease, also explored αT’s potential for cancer prevention but found no reduction in cancer incidence or mortality. The *Supple’ mentation en Vitamines et Miéraux Antioxydants* (SUVIMAX) study [58] in France, which tested combined antioxidant supplementation, showed mixed results, with no clear benefit in reducing cancer risk across the entire cohort, though some protective effects were observed in subgroups with specific risk factors. The Women’s Health Study (WHS) [59] in healthy women, the Heart Outcomes Prevention Evaluation (HOPE) and HOPE-The Ongoing Outcomes (HOPE-TOO) [60] as well as the Selenium and vitamin E Cancer Prevention Trial (SELECT) [61], all showed no significant impact of αT on cancer prevention. Lastly, the Physician’s Health Study II (PHSII) [62] similarly found no evidence of benefit in reducing cancer incidence or mortality among older men. These trials collectively highlight that the results have been inconsistent, with only the Linxian, ATBC, and SUVIMAX studies showing some benefit, typically in populations with specific deficiencies or risk factors. In contrast, animal studies have also yielded mixed results, and more recent studies suggest that other forms of vitamin E, such as γT and δT, may offer more promising anticancer effects, potentially due to their distinct biological properties compared to αT.

#### 3.2.2. Non-α-Tocopherol in Cancer Prevention

Despite the fact that most studies in cancer prevention have focused exclusively on the αT form of vitamin E in the past, recent studies by others and our group have shown that other forms of vitamin E, including γT, δT, γTE and δTE, appear to have unique and stronger anticancer activities (Table 1).

γT, the major form of vitamin E in many plant seeds and in the US diet, and the second most common tocopherol in human serum, has unique and important properties for cancer prevention and therapy that are not shared by αT. First of all, as we already mentioned in Section 2.3, γT exerts anti-inflammatory activities, which are known to play important roles in cancer prevention. Specifically, our previous studies have shown that γT and γ-CEHC, but not αT, exert anti-inflammatory activities by inhibition of prostaglandin E_2_ (PGE_2_) synthesis, which occurred through COX-catalyzed reaction, in activated macrophages and epithelial cells and by inhibition of 5-LOX-catalyzed synthesis of leukotriene B_4_ (LTB_4_) in carrageenan-induced inflammation in rats [28,39]. Furthermore, RRR-γT, but not RRR-αT induced apoptosis in multiple colon cancer cell lines, but not in normal colon cells [40]. In addition to a number of cancer cell studies, several animal studies were conducted to investigate whether these anticancer effects of γT are also translated into in vivo animal models. Yu et al. reported that γT supplementation, but not αT, significantly reduced breast tumor growth compared with control diet group in xenograft mouse models [63,64]. γT, but not αT, also suppressed tumor progression, along with activation of caspase-3 and -7 in the ventral lobe in a transgenic prostate cancer rat model [65]. Our group recently demonstrated that γT significantly attenuated moderate colitis and suppressed inflammation-promoted colon tumorigenesis [66]. Furthermore, several studies investigated the anticancer effects of γT in vivo using γT-rich mixed tocopherols (γ-TmT). γ-TmT supplementation as well as γT or δT produced a significant inhibition of azoxymethane (AOM)-induced aberrant crypt foci (ACF; a precancer lesion) in the colon of rats [67,68]. Another study was conducted to test the effects of γ-TmT against colon cancer, and in this study, γ-TmT suppressed AOM/dextran sodium sulfate (DSS)-induced tumorigenesis in CF-1 mice [69]. Besides colon cancer, γ-TmT was also effective in suppression of breast tumorigenesis in preclinical rat models [70,71,72]. Moreover, in a human study, Helzlsouer et al. conducted a nested case-control study to investigate the associations of αT, γT, and selenium with incidence of prostate cancer. They observed that higher plasma γT concentrations were associated with a statistically significant lower risk of developing prostate cancer. Interestingly, this protective association against prostate cancer was found with only γT, not with αT or selenium [73].

Besides tocopherols, recent studies suggested that tocotrienols, especially γTE and δTE, appear to have potent anticancer effects in various cancer cells. γTE, which is an abundant vitamin E form in palm oil, has been shown to have strong anti-inflammatory activities by inhibition of TNFα-stimulated NF-κB, TAK1 and JNK activation via sphingolipid modulation, ER stress induction, and A20 upregulation [30]. γTE inhibited cell proliferation and induced apoptosis in human colon cancer cells, by the mechanisms of cell cycle arrest, an increase of Bax/Bcl-2 ratio, and activation of caspase-3 [50]. Our group also showed that γTE induced apoptosis and autophagy by interrupting sphingolipid modulation in prostate and breast cancer cells [41,52]. Several studies have shown that γTE and δTE significantly inhibited cell proliferation and induced apoptosis in both estrogen receptor-negative MDA-MB-435 and estrogen receptor-positive MCF-7 human breast cancer cells [44,45,46,74]. Consistent with the results in cancer cell culture studies, γTE and δTE showed effective anticancer properties in several in vivo animal studies. For instance, several studies have shown that γTE supplementation significantly inhibited prostate tumor growth in xenograft models [52,75,76]. γTE also showed these anticancer properties against pancreatic cancer, which generally shows resistance to chemotherapy, by inhibition of tumor growth and sensitization it to gemcitabine [77]. Interestingly, Hiura et al. reported that both γTE and δTE supplementation significantly delayed liver tumor growth, by particularly being accumulated in tumors, not in normal tissues [78]. In addition, δTE was observed to show potent tumor antiangiogenic potential compared with αT [79].

**Table 1:** In vitro anticancer effects and mechanisms of non-α-tocopherol forms of vitamin E.

Type of Vitamin E	Cell Type/Origin	Results	Mechanisms	Ref.
γT	Human colon cancer cells (SW480, HCT-15, HCT-116, HT-29)	Antiproliferation and cell stress and death	Apoptosis ↑ (cleavage of PARP, caspase 3, 7, and 8)	[40]
	Human LNCaP prostate, MCF-7 breast cancer cells	Antiproliferation and cell death	Apoptosis ↑ (cleavage of PARP, caspase 3 and 9, Cytochrome C release) via sphingolipid modulation	[41,42,43]
δT	Human prostate cancer cells (PC-3, DU145, LNCaP)	Antiproliferation and cell death	Cell cycle arrest, apoptosis ↑, AKT phosphorylation ↓	[51,80]
γTE	Human prostate cancer cells (PC-3, LNCaP)	Cell death	Induction of apoptosis and autophagy ↑ via sphingolipid modulation	[52]
	Human breast cancer cells (MCF-7, MDA-MB-231)	Antiproliferation and cell death	ER stress-mediated apoptosis, autophagy ↑	[81,82]
	Neoplastic mammary epithelial cells	Inhibition of cell proliferation and induction of apoptosis	Akt phosphorylation and NF-κB activity ↓ PI3K/PDK-1 ↓	[53,54]
	Human MCF-7 breast, HCT116 colon cancer cells	Antiproliferation and cell death	Apoptosis, autophagy, cell death ↑ via sphingolipid modulation	[41,83]
δTE	Human melanoma cells (BLM, A375)	Antiproliferation and cell death	ER stress-mediated apoptosis ↑	[47]
	Human prostate cancer cells (PC3, DU145)	Cytotoxic/proapoptotic effect	ER stress, apoptosis, autophagy, paraptosis ↑	[48]
	Human pancreatic cancer cells (MiaPaCa-2, PANC-1, SW1990)	Cell death and sensitization to TRAIL-induced apoptosis	Caspase-8-dependent apoptosis ↑ via c-FLIP degradation	[49]

γ- and δ-tocopherol (γT and δT) and γ- and δ-tocotrienol (γTE and δTE). AKT, protein kinase B; ER, endoplasmic reticulum; NF-κB, nuclear factor kappa-light-chain-enhancer of activated B cells; PARP, poly(ADP-ribose) polymerase; PI3K, phosphatidylinositol 3-kinase. ↑, upregulation; ↓, downregulation.

#### 3.2.3. 13′-Carboxychromanol in Cancer Prevention

Sontag and Parker first discovered cytochrome P450 ω-hydroxylase pathway of tocopherol catabolism and identified all key intermediates including long- and short-chain carboxychromanols and final metabolite, 3′-carboxychromanol in human hepatocyte [12]. Consistently, our group identified these intermediates after treatment with γT and δT in human A549 cells, and discovered that in parallel with β-oxidation, sulfation of intermediate metabolites takes place. Importantly, these sulfated or non-sulfated long-chain carboxychromanols were found in rat plasma and liver upon vitamin E supplementation [13]. We also observed the similar metabolism of γTE in human A549 cells and in rats [84]. Bardowell et al. revealed the presence of all six carboxychromanol metabolites and 13′-OH in fecal samples from mice fed with 800 mg/kg body weight γT and δT for 12 weeks. They also found similar findings in fecal material from an adult male supplemented with 400 mg/kg/day γT for 14 days [85].

Recently, we began to investigate the biological activities of these vitamin E metabolites and demonstrated that long-chain carboxychromanols have stronger anti-inflammatory activities than their unmetabolized vitamin E forms. Particularly, δT-13′-COOH, a long-chain carboxychromanol from δT, has been shown to exert anti-inflammatory effects by showing much more potent inhibiting actions on COX-1/COX-2 and 5-LOX activities than short-chain carboxychromanols and unmetabolized vitamin E precursors [31,32]. Moreover, Birringer et al. investigated the biological activities of long-chain carboxychromanols synthesized from garcinoid acid, a δTE derivative extracted from the African bitter nut *Garcinia kola*, in human HepG2 hepatocellular liver carcinoma cells. 13′-COOHs from αT and δT have shown to induce mitochondria-mediated apoptosis with increased mitochondrial ROS formation and reduced mitochondrial membrane potential [86]. Although, the results of these studies show that vitamin E metabolites, especially 13′-COOHs exert potential anticancer properties, they may contribute to the beneficial effects of vitamin E forms in vivo. However, the effects and the underlying mechanisms by which 13′-COOHs exert these beneficial effects are not completely understood yet, which warrants further investigation.

## 4. Sphingolipid Mechanisms and Cancer Prevention: Role of Vitamin E and Its Metabolites

### 4.1. Modulation of Sphingolipid Metabolism by Vitamin E and Its Metabolites in Cancer Cells

Several studies have shown that vitamin E forms and their metabolites, 13′-COOHs have anti-proliferative and pro-death effects via modulating sphingolipid metabolism. γT was initially found to inhibit proliferation and induce apoptosis in human prostate cancer cells, but not in normal prostate epithelial cells, by interrupting sphingolipid metabolism, specifically by accumulating dihydrosphingosine (dhSph) and dihydroceramides (dhCer) [42]. Follow-up studies also reported that γT induced apoptosis in human breast cancer cells by increasing cellular ceramides (Cer) and dhCer levels and activating the c-Jun N-terminal kinase (JNK)/CCAAT/enhancer-binding protein homologous protein (CHOP)/death receptor-5 (DR5) proapoptotic signaling pathway [41,43]. Moreover, γTE was found to be more potent than γT in promoting cancer cell death through the modification of sphingolipid metabolism [83].

Additionally, 13′-COOH derived from δT or δTE inhibited pro-inflammatory enzymes and induced apoptosis and autophagy in human colon cancer cells by modulating sphingolipid metabolism [87]. Specifically, treatment with γTE and 13′-COOH from δT or δTE induced increases in intracellular dhCers and dhSph in the de novo sphingolipid pathway in cancer cells over a short period via the inhibition of DEGS activity. However, longer time treatment with these compounds elevated intracellular Cers, suggesting that sphingomyelin hydrolysis by sphingomyelinase is likely activated by these compounds. These data indicate that modulation of sphingolipid metabolism likely contributes to the anticancer effects of natural forms of vitamin E and 13′-COOHs.

### 4.2. De Novo Sphingolipids Biosynthesis

**Figure 4:**
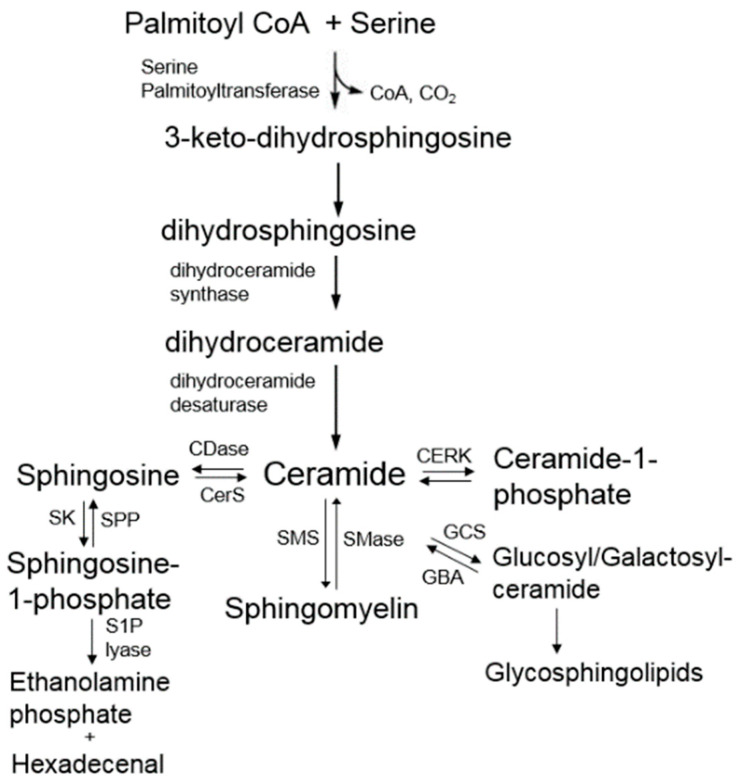
Sphingolipid metabolism. De novo biosynthesis pathway of sphingolipids, the production of complex sphingolipids from ceramide, the degradation of ceramide to sphingosine, the formation of sphingosine-1-phosphate (S1P), and the clearance of S1P by S1P lyase. SMS, sphingomyelin synthase; SMase, sphingomyelinase; CERK, ceramide kinase; GCS, glucosylceramide synthase; GBA, acid glucocerebrosidase; SK, sphingosine kinase; SPP, S1P phosphatase.

**Figure 5:**
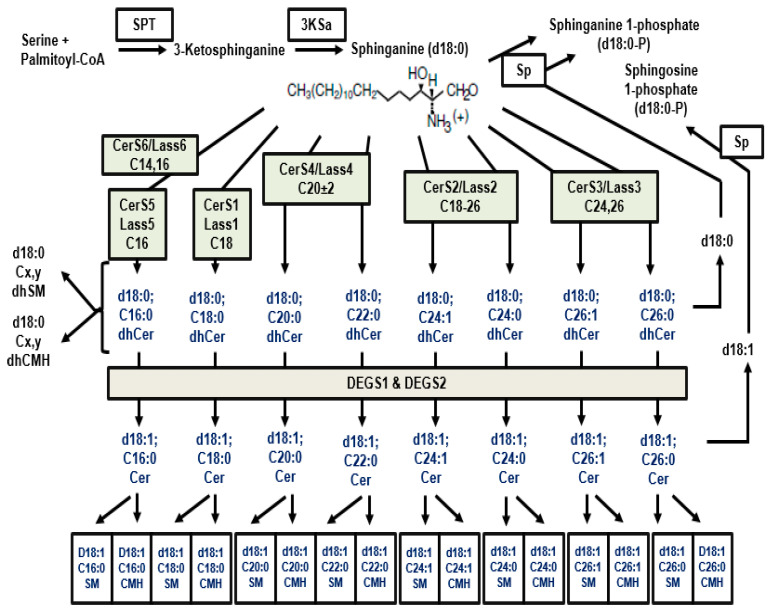
De novo biosynthesis of sphingolipids highlighting the synthesis of many ceramides with different fatty acid chain length. Biosynthesis of sphinganine, which can be acylated by the CerS/Lass gene products with their specific fatty acyl-CoA preference. Each dhCer can be desaturased to the corresponding Cer species, followed by the production of more complex sphingolipids. Cer, ceramide; CerS, ceramide synthase; dhCer, dihydroceramide; DEGS, dihydroceramide desaturase; SM, sphingomyelin.

The de novo sphingolipid biosynthesis (Figure 4 and Figure 5) begins in the endoplasmic reticulum, where serine and palmitoyl-CoA are condensed by serine palmitoyltransferase (SPT) to form 3-keto-dihydrosphingosine, which is reduced to dhSph. This is followed by acylation by dihydroceramide synthase (CerS), which generate dhCer. Finally, dihydroceramide desaturase (DEGS) introduces a 4,5-*trans* double bond to convert dhCer into Cer [88].

#### 4.2.1. Serine Palmitoyltransferase (SPT)

SPT catalyzes the initial condensation of serine and palmitoyl-CoA to produce 3-keto-dihydrosphingosine. This enzyme is regulated by the availability of substrates and can be inhibited by compounds such as myriocin. SPT is crucial for de novo sphingolipid biosynthesis, and several inhibitors, both natural and synthetic, have been identified [89,90].

#### 4.2.2. (Dihydro)ceramide Synthase (CerS)

CerS enzymes acylate dhSph to form various dhCer species, with different CerS isoforms having distinct preferences for fatty acyl-CoA chain lengths. For example, CerS1 primarily produces C18-Cer, while CerS5 and CerS6 predominantly generate C16-Cer species [91,92]. Inhibition of CerS can occur through compounds such as fumonisin B1, which blocks enzyme activity by competing with dhSph and fatty acyl-CoA [93].

#### 4.2.3. Dihydroceramide Desaturase (DEGS)

DEGS (Dihydroceramide Δ4-desaturase) is responsible for the final step in de novo sphingolipid biosynthesis, converting dhCer to Cer by inserting a 4,5-trans double bond. DEGS utilizes molecular oxygen to add a hydroxyl group at the C4 position of dhSph and, with NADP or NADPH, catalyzes dehydration to form the double bond between C4 and C5 of dhCer. Two forms of DEGS, DEGS1 and DEGS2, have been identified. DEGS1 predominantly acts as a Δ4-desaturase, while DEGS2 has both Δ4-desaturase and C-4 hydroxylase activities. DEGS is located in the ER membrane, where it interacts with dhCer species. Its activity is influenced by factors such as the length of the sphingoid base alkyl chain and the stereochemistry of dhSph. Additionally, DEGS activity is inhibited by compounds like cyclopropene-containing Cer and GT-11 [94,95,96,97].

### 4.3. Synthesis of Complex Sphingolipids

Cer produced in the endoplasmic reticulum is transported to the Golgi for the synthesis of complex sphingolipids [98]. This process involves vesicular transport or the Cer transfer protein (CERT), which delivers Cer for sphingomyelin (SM) synthesis. Glucosyl- and galactosyl-Cer are synthesized by glucosyl-Cer synthase and Cer galactosyltransferase, respectively. SM is produced by SM synthases (SMS), with SMS1 and SMS2 localized to the trans-Golgi. SMS transfers a phosphocholine headgroup from phosphatidylcholine to Cer to produce SM and diacylglycerol (DAG) [99,100,101]. The activity of SMS is inhibited by compounds like D609 [102]. Complex sphingolipids, including SM, are delivered to the plasma membrane, where they play important structural and functional roles [103].

### 4.4. Catabolism of Complex Sphingolipids and Ceramide

SM can be hydrolyzed by sphingomyelinases (SMases) to produce Cer and phosphocholine. Acid SMases, found in lysosomes, and neutral SMases, located in the bilayer, both metabolize SM [104,105]. SMase activation is linked to factors like oxidative stress [105], serum starvation [106], and treatment with vitamin D [107]. Cer is also broken down by ceramidases (CDases) to form sphingosine, which can be phosphorylated to sphingosine-1-phosphate (S1P) by sphingosine kinases (SphK). The final step in sphingolipid catabolism involves the degradation of S1P by S1P lyase [104].

### 4.5. Bioactive Sphingoid Bases and Their Roles in Cell Growth, Survival, and Death

Sphingolipids are diverse groups of lipids that play a variety of essential roles as components of cell membrane structure and cell signaling molecules, thus affecting on the mammalian development and physiology. Moreover, dysregulated sphingolipids metabolism is known to occur in some diseases such as cancer, diabetes, atherosclerosis, and neurodegenerative diseases. Over the past two decades, several sphingolipids metabolites, such as sphingosine, Cer, and S1P were defined as bioactive lipid messengers and regulatory molecules, and many researchers have begun to investigate their various roles and functions. These metabolites are now clearly known to play critical roles in regulating various cellular events including differentiation, proliferation, apoptosis, and autophagy [108,109]. However, as the sphingolipid metabolism is complex and dynamic processes, and the produced metabolites have different or opposite functions, their relative levels and balance between each other are important for the regulation of survival and death within the cells. For instance, while Cer has antiproliferative and proapoptotic properties, S1P involves in cellular proliferation and survival. Thus, a model has been previously proposed in which the equilibrium between these two molecules, the ‘Cer/S1P rheostat’, could determine cell fate [110]. Besides this model, recent studies have suggested that sphingolipid metabolites are interconvertible and each metabolite has its distinct functions. For example, Cer can be further metabolized to sphingosine by ceramidase, which has been shown to induce apoptosis in many cell types. This sphingosine can be further phosphorylated to form S1P by sphingosine kinase. In addition, Cer can be generated from SM via SMases. Therefore, now it is important to understand this complexity of sphingolipid metabolism and specific enzymes that are involved in the pathway, and define the roles and regulation of each metabolite by the application of the comprehensive ‘sphingodynamic’ model. This present review will focus on the roles of several bioactive sphingolipid metabolites and their regulation (Table 2).

#### 4.5.1. Sphingosine

Sphingosine, generated from Cer by ceramidase (CDase), can be phosphorylated to form sphingosine-1-phosphate (S1P) by sphingosine kinase. Sphingosine is produced during apoptosis and can induce apoptosis in many cell types [110,111]. It inhibits MAPKs (Erk-1/2) and activates JNK/p38, leading to caspase activation and mitochondrial apoptosis. Sphingosine also downregulates the PI3K-Akt pathway, contributing to apoptosis [110].

**Table 2:** Sphingolipids as regulators of cancer cell death.

Type of Sphingolipid	Type of Cancer/Model	Results	Mechanisms	Ref.
Sphingosine 1-phosphate	HEL cells	Cellular proliferation, differentiation, migration, and mitogenesis, and survival	SphK activity ↑ (by growth factors, cytokines, GPCRs), ERK-1/2, PLD ↑	[112]
	Jurkat T lymphocytes, HL60, U937 leukaemia cells	Antiapoptosis	Caspases, JNK ↓, PKC ↑	[112,113]
Sphingosine	Leukemic cells, solid cancer cell lines	Proapoptosis	PKC, ERK1/2, PI3K-Akt ↓, JNK/p38, caspases activation ↑	[110]
	Human colon cancer cells (HT-29, HCT-116)	Growth inhibition and induction of cell death	Cell cycle arrest, apoptosis	[111]
Ceramide	Various cancer cells	Antiproliferation and proapoptosis	Mitochondrial pathway, caspase activation ↑	[114,115]
	Human colon cancer HT-29 cells	Autophagic cell death	PKB ↓, beclin1 ↑	[116]
	Human colon cancer cells (HT-29, HCT-116)	Growth inhibition and induction of cell death	Cell cycle arrest, apoptosis	[111]
	Human corneal epithelial, breast cancer MCF-7 cells	Anti-inflammation	Cytokine production ↓	[117,118]
Dihydrosphingosine	Colon, prostate cancer, and leukemic cells	Apoptosis, autophagy, and cell death	PI3K-Akt ↓,	[42,52,111,119,120]
	Various cancer cells	Autophagy	PKCs, PI3K ↓	[121]
Dihydroceramide	Prostate cancer cells, neuroblastoma cells	Cell cycle arrest, apoptosis, oxidative stress	DEGS inhibition	[42,122,123]

DEGS, dihydroceramide desaturase; ERK-1/2, extracellular signal-regulated kinase-1/2; GPCRs, G protein-coupled receptors; JNK, c-Jun-N-terminal kinase; PI3K, phosphatidylinositol 3-kinase; PKB, protein kinase B; PKC, protein kinase C; SphK, sphingosine kinase; PLD, phospholipase D. ↑, upregulation; ↓, downregulation.

#### 4.5.2. Ceramide

Cer, synthesized via de novo synthesis or sphingomyelin (SM) catabolism, regulates various cellular events such as cell death, senescence, and inflammation. It can activate protein phosphatases (CAPPs) and protein kinases, influencing apoptosis by dephosphorylating targets like Rb, PKCα, AKT, and Bcl-2 [98,124,125]. Cer also induces apoptosis through the mitochondrial pathway and by downregulating the PI3K-Akt pathway [111,114]. In addition, Cer has been linked to autophagy induction and anti-inflammatory effects. Recent research indicates that Cer species with different fatty acid chain lengths have distinct bioactivities, influencing cancer cell growth and survival. Specifically, while C16:0-Cer plays a prosurvival role by preventing apoptosis, C18:0-Cer exhibits proapoptotic effects and can also induce lethal autophagy independent of apoptosis, particularly in HNSCC cell lines. These findings suggest that C16:0-Cer and C18:0-Cer, generated by CerS1 and CerS5 respectively, play opposite roles in determining cancer cell fate, either promoting survival or triggering cell death [126,127].

#### 4.5.3. Dihydrosphingosine and Sphingosine-1-Phosphate

DhSph, an upstream intermediate in sphingolipid biosynthesis, induces apoptosis in various cancer cells and immune cells. Notably, γ-tocopherol (vitamin E) and safingol (a synthetic dhSph analog) promote dhSph accumulation, leading to apoptosis and autophagy through PI3K and PKC inhibition. Fumonisin B1, a CerS inhibitor, also causes dhSph accumulation and toxicity [52,111,128].

In contrast, sphingosine-1-phosphate (S1P), produced by sphingosine kinase (SphK), plays a pro-survival role, promoting cell proliferation, migration, and resistance to apoptosis. S1P signaling via G protein-coupled receptors (GPCRs) regulates various cellular responses, including differentiation and metastasis. Elevated S1P levels are associated with cancer cell resistance to death and may promote angiogenesis and inflammation, contributing to tumor progression [112,129,130,131].

#### 4.5.4. Dihydroceramide

DhCer is a sphingolipid metabolite converted to Cer via DEGS. Initially thought to be an inactive precursor, dhCer has now been recognized as bioactive. Early studies showed dhCer did not induce apoptosis like Cer, but recent research revealed that dhCer inhibits Cer channel formation in mitochondria, blocking apoptosis [132]. Advanced LC-MS/MS techniques have shown that compounds like 4-hydroxy phenylretinamide (4-HPR) increase dhCer, not Cer, by inhibiting DEGS [108,133,134].

DhCer is involved in various cellular responses, including cell cycle arrest [122], apoptosis [41,42,52], autophagy [52,108,123], and oxidative stress [135]. Compounds such as γ-tocopherol (γT) [52], resveratrol [123], and fenretinide [136] elevate dhCer levels, promoting apoptosis and autophagy. γT, in particular, induces apoptosis in prostate cancer cells by increasing dhCer and dhSph [52]. Inhibition of DEGS, either through natural compounds or oxidative stress, leads to dhCer accumulation and affects cell proliferation. Recent studies have also shown γTE, an analog of γT, induces apoptosis and autophagy in cancer cells by increasing dhCer levels and inhibiting NF-κB [30,41,52].

## 5. Conclusions

Clinical studies on αT for cancer prevention have often yielded disappointing results, leading to the exploration of other non-α forms of vitamin E and their metabolites. While αT has long been studied due to its antioxidant properties, recent evidence suggests that other forms of vitamin E, such as γT, δT, and their metabolites, may have stronger anticancer potential. These compounds exhibit a broad range of bioactivities beyond antioxidant effects, including anti-inflammatory properties, modulation of bioactive lipids, and regulation of key signaling pathways, such as NF-κB, STAT3, and PI3K/Akt. Particularly noteworthy is the role of sphingolipid metabolism in cancer prevention. Sphingolipids are crucial bioactive lipids that influence key cellular processes such as cell growth, differentiation, and apoptosis. Non- αT forms of vitamin E, including γT, δT, and their metabolites like 13′-carboxychromanol, have been shown to modulate sphingolipid metabolism in ways that contribute significantly to their anticancer effects. These include inducing cell death through mechanisms like antiproliferation, apoptosis, and autophagy with an emphasis on enhancing Cer levels, a well-known pro-apoptotic molecule. Furthermore, studies suggest that the ability of these non-αT forms to regulate other lipid-based pathways, including sphingomyelinase activity and Cer synthase, enhances their cancer-fighting properties. Although clinical trial results for αT have been inconsistent, research on the non-αT forms of vitamin E suggests that their impact on sphingolipid pathways could provide a promising new avenue for cancer prevention and therapy. Given these diverse mechanisms, non-αT forms and their metabolites hold considerable therapeutic potential, and future experimental and epidemiological studies are needed to fully elucidate their roles and efficacy in cancer prevention and treatment. These findings also underscore the need for more targeted studies that explore the mechanistic pathways through which these compounds influence cancer cell biology.

## 6. Future Perspectives

Future research on vitamin E should explore several important areas to better understand its therapeutic potential in cancer prevention and treatment. A key direction is the investigation of various non-αT forms and their metabolites, which show promising anticancer effects. While αT is the most abundant form and has a limited anticancer effect, non-αT forms of vitamin E undergo more extensive metabolism, producing bioactive metabolites with significant anticancer properties. These vitamin E isoforms, through both their antioxidant and non-antioxidant activities, can modulate key cancer-related pathways such as inflammation, bioactive lipid regulation, and sphingolipid metabolism, contributing to cell growth inhibition, apoptosis, and autophagy in cancer cells.

In particular, understanding how non-αT and their metabolites affect sphingolipid metabolism in rare and hard-to-treat cancers, such as pancreatic cancer, ovarian cancer, and brain cancer, could reveal novel therapeutic strategies.

Moreover, the combination of vitamin E isoforms with existing cancer therapies, such as chemotherapy or immunotherapy, could enhance treatment efficacy while reducing side effects. To maximize their therapeutic potential, novel drug delivery systems are crucial. Since non-α forms of vitamin E undergo significant metabolism, developing targeted delivery systems, such as nanoparticles or liposomes, could help direct active metabolites to tumor sites, improving bioavailability and effectiveness. Additionally, incorporating personalized medicine approaches based on genetic differences in vitamin E metabolism could allow for more tailored treatments, optimizing therapeutic outcomes for individual patients. In conclusion, further experimental and clinical studies are necessary to fully unlock the therapeutic potential of vitamin E and its metabolites, particularly in the context of sphingolipid metabolism modulation and cancer therapy.
